# Elicitation as a tool to improve the profiles of high‐value secondary metabolites and pharmacological properties of *Hypericum perforatum*

**DOI:** 10.1111/jphp.12743

**Published:** 2017-05-19

**Authors:** Preeti Shakya, Gregory Marslin, Karthik Siram, Ludger Beerhues, Gregory Franklin

**Affiliations:** ^1^ Department of Integrative Plant Biology Institute of Plant Genetics of the Polish Academy of Sciences Poznań, Wielkopolska Poland; ^2^ Chinese‐German Joint Laboratory for Natural Product Research Qinling‐Bashan Mountains Bioresources Comprehensive Development C.I.C. College of Biological Science and Engineering Shaanxi University of Technology Hanzhong Shaanxi China; ^3^ Department of Pharmaceutics PSG College of Pharmacy Coimbatore Tamil Nadu India; ^4^ Institute of Pharmaceutical Biology Technische Universität Braunschweig Braunschweig Germany

**Keywords:** elicitation, *Hypericum perforatum*, *in vitro* cultures, nanoparticles, plant secondary metabolism

## Abstract

**Objectives:**

In this review, we aim at updating the available information on the improvement of the *Hypericum perforatum* L. (*Hypericaceae*) phytochemical profile and pharmacological properties *via* elicitation.

**Key findings:**

*Hypericum perforatum* seedlings, shoots, roots, calli and cell suspension cultures were treated with diverse elicitors to induce the formation of secondary metabolites. The extracts of the elicitor‐treated plant material containing naphthodianthrones, phloroglucinols, xanthones, flavonoids and other new compounds were quantitatively analysed and tested for their bioactivities. While hypericins were mainly produced in *H. perforatum* cultures containing dark nodules, namely shoots and seedlings, other classes of compounds such as xanthones, phloroglucinols and flavonoids were formed in all types of cultures. The extracts obtained from elicitor‐treated samples generally possessed better bioactivities compared to the extract of control biomass.

**Summary:**

Although elicitation is an excellent tool for the production of valuable secondary metabolites in *H. perforatum* cell and tissue cultures, its exploitation is still in its infancy mainly due to the lack of reproducibility and difficulties in scaling up biomass production.

## Introduction

*Hypericum perforatum* L. (*Hypericaceae*) known as St John's wort is an important medicinal plant. Extracts, infusions and decoctions have been used in the treatment of various ailments since ancient times. A number of pharmacological studies and clinical trials have shown that *H. perforatum* extracts possess an astounding array of pharmacological properties including antidepressant, anti‐inflammatory, antiviral, anticancer and antibacterial activities. In particular, *H. perforatum* extracts are as efficient as their drug counterparts like fluoxetine (Prozac), sertraline (Zoloft) and other leading antidepressant drugs in the treatment of depression. Nowadays, it is a widely used medicinal plant for the treatment of mild and moderate depression,^1^ prescribed for this indication in some EU countries, while sold in the United States as herbal supplement over the counter.^2^

**Figure 1:**
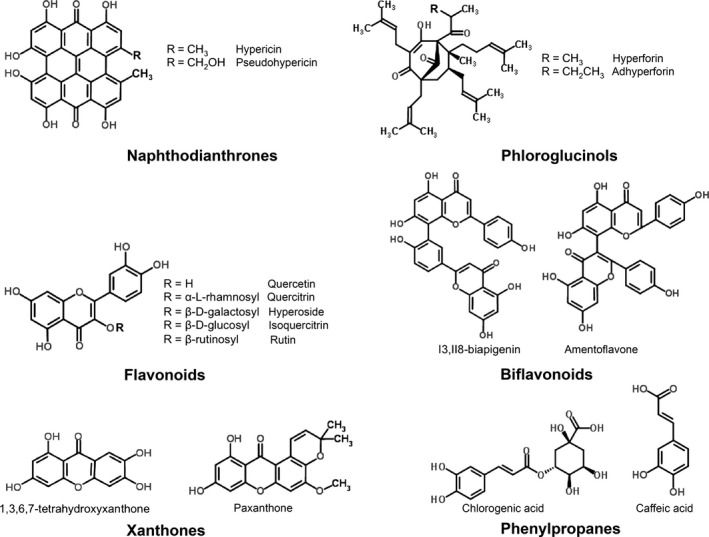
Major classes of secondary metabolites found in *Hypericum perforatum*.

In addition to its efficacy in the treatment of neurological disorders, studies suggest that this herb may be useful in treating cancer, inflammation‐related disorders and bacterial and viral diseases.^3^, ^4^, ^5^, ^6^, ^7^ These medicinal properties are related to the composition of the secondary metabolites present in the extract, particularly hypericins, hyperforins, flavonoids, xanthones and other valuable compounds.^8^ Phytochemical characterization of *H. perforatum* revealed the presence of various classes of compounds, including naphthodianthrones (hypericin and pseudohypericin), prenylated acylphloroglucinols (hyperforin and adhyperforin), flavonoids (quercetin, hyperoside, rutin, catechin and isoquercitrin), biflavones (amentoflavone, biapigenin), phenolic compounds (chlorogenic acid, tannic acid and caffeic acid), xanthones (1,3,6,7‐tetrahydroxyxanthone) and essential oil rich in sesquiterpenes^9^, ^10^, ^11^ (Figure 1).

Currently, *H. perforatum* is one of the top‐selling herbal medicines worldwide. Because of its reputed medicinal values, this species is included in the Pharmacopoeias of several countries including Europe and USA. According to the U.S. and European Pharmacopoeias, the crude drug consists of the dried flowering tops or the aerial parts of the plant. In order to meet the ever‐increasing demands of the pharmaceutical industry and to obtain quality biomass, *H. perforatum* is cultivated in many countries. Plants growing in the field conditions are generally exposed to biotic and abiotic challenges, which can affect the phytochemical composition. For example, *H. perforatum* plants obtained from different geographical regions, seasons, soil conditions significantly differ in their phytochemical profile.^12^, ^13^, ^14^ As the pharmacological potential of *H. perforatum* extracts is mainly determined by its phytochemical composition and ratios between important compounds, such changes may affect the treatment efficacy of the extracts.^15^, ^16^ *In vitro* cultures grown under controlled conditions can overcome these issues and have been emerged as an attractive alternative to field cultivation.^17^, ^18^

Today, enhancing the contents of important bioactive molecules and producing novel compounds are major aspects of *H. perforatum* biotechnology. Developments in plant cell culture systems and molecular biology offered many ways to improve the production of compounds such as cell line selection, cell immobilization, permeabilization, precursor feeding, product secretion, biotransformation, metabolic engineering, bioreactor engineering, synthetic biology and elicitation.^19^, ^20^, ^21^ Among all, elicitation emerges as an attractive strategy for enhancement of secondary metabolite production in plant species like *H. perforatum*, in which the application of metabolic engineering or synthetic biology tools remains difficult due to the lack of proficient transformation methods and genetic information about biosynthetic pathways.^22^ Hence, production of secondary metabolites *via* elicitation using various *in vitro* culture systems is of great interest in *H. perforatum* research.

Significant amounts of data on the manipulation of *H. perforatum* secondary metabolism *via* elicitation have been accumulated in the literature in the recent years. However, a consolidated account or a critical analysis of these published data is not available to the best of our knowledge. Here, we holistically review the available information on the elicitation of *H. perforatum* secondary metabolism, envisage the potential application of nanoparticles as elicitors and discuss how elicitation‐mediated improvement of the *H. perforatum* secondary metabolite profile may be exploited for drug discovery.

## The basis of elicitation

Plants have to defend themselves against any threats posed by their environment, such as pathogen attack, herbivory, drought, salinity, exposure to UV radiation. Plants perceive such danger signals through their receptors and sensors and activate defence responses to counteract against these stresses.^23^, ^24^, ^25^, ^26^ The responses include secondary metabolism.^27^ For instance, plants recognize pathogen‐derived elicitors through receptors bound to the plasma membrane and activate, as a defence response, the production of low‐molecular‐weight antimicrobial compounds. While these phytoalexins are synthesized by and accumulated in plants only after exposure to pathogenic microbes, phytoanticipins are either pre‐existing or synthesized after the microbial attack solely from pre‐existing constituents of the plant.^28^ In addition to their role in plant defence response, these compounds often possess important pharmacological properties.^29^, ^30^ The ability of plants to counteract with biotic and abiotic stresses *via* mobilizing their secondary metabolism is the central dogma of elicitation.

An elicitor can be an environmental factor or a signal molecule that activates a signal‐transduction cascade, which mediates the expression of genes related to the biosynthesis of secondary metabolites in the biotechnological point of view. Elicitors are mainly classified into three categories based on their origin, namely biological, chemical and physical triggers. The biological elicitors are mainly components of microbial cell walls (chitin, chitosan and glucans) and carbohydrates such as poly‐ and oligosaccharides derived from plant cell walls (pectin, pectic acid, and cellulose). Poly‐ and oligosaccharides are the most studied signalling molecules for elicitation pathways because these compounds can effectively induce similar plant defence responses to pathogen invasion.^31^ Upon elicitation, a series of metabolic changes are systemically initiated throughout the plant to activate the plant's innate immune system^32^, ^33^ and also to prime the plant for stress challenge.^34^ Furthermore, the plant defence signalling compounds such as salicylic acid (SA), jasmonic acid (JA), methyl jasmonate (MeJA) and nitric oxide which mediate the defence response can also serve as elicitors and their ability to induce secondary metabolism is well‐documented.^8^, ^35^, ^36^, ^37^, ^38^, ^39^ Inorganic agents like heavy metals, metal ions and metal oxides can act as chemical elicitors of plant secondary metabolism.^40^, ^41^, ^42^ Physical components like cold shock, UV, ozone, osmotic and water stress also induce enzymatic activity and secondary metabolism.^43^

The mechanism of elicitation is vastly complex because of thousands of intertwined events. Additionally, all these events fluctuate with the origin, specificity and concentration of elicitors, stage of the growth cycle and nutritional uptake of plants, physiochemical environment of the interaction etc. Although it is difficult to propose a universal model for the elicitation mechanism, calcium flux, reactive oxygen species (ROS) burst and mitogen‐activated protein kinase (MAPK) phosphorylation are the initial events triggered in most of the elicitor–plant cell interactions.^44^ Later events like activation of signalling pathways and activation of transcription factors leading to the induction of plant secondary metabolism are also well‐documented.^45^, ^46^, ^47^

**Figure 2:**
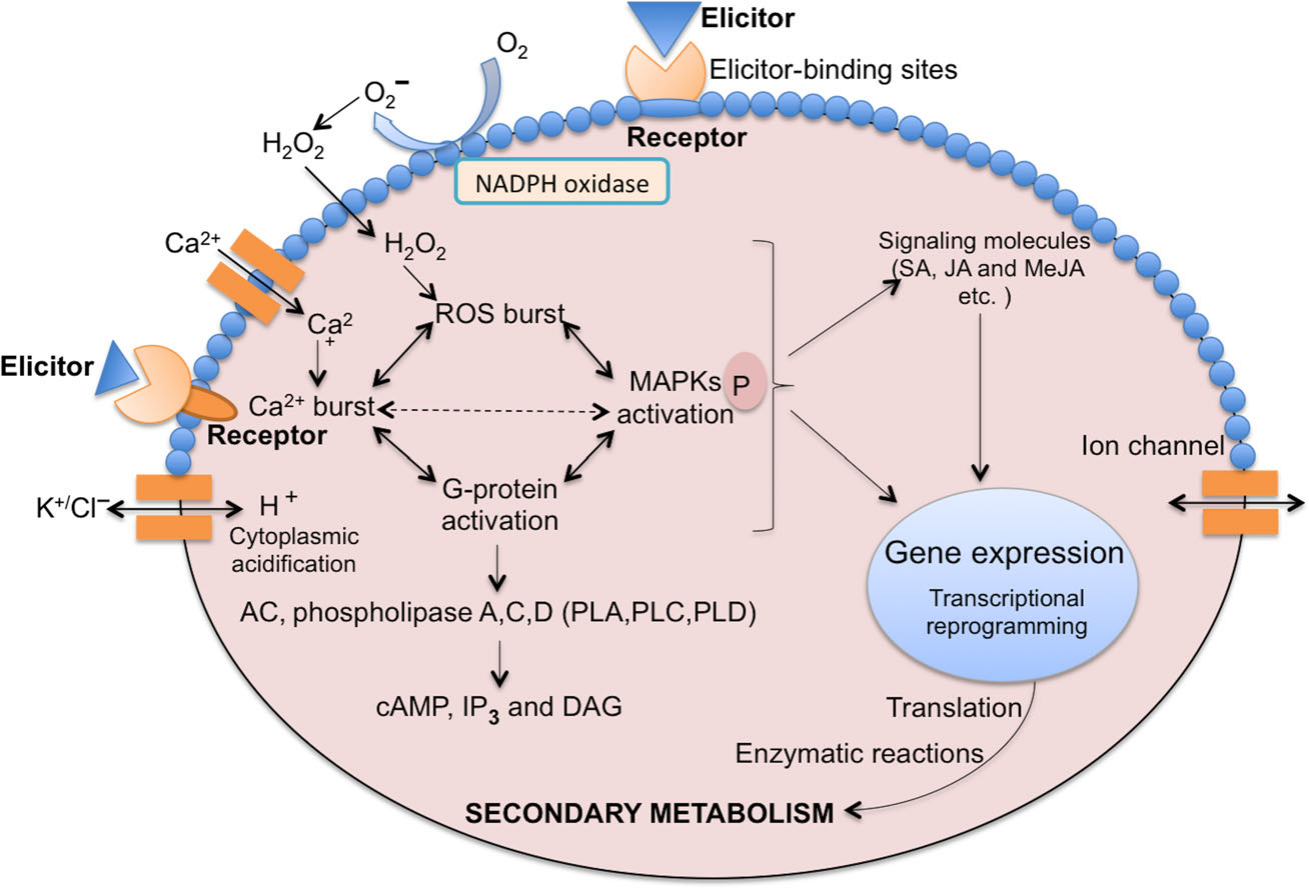
The molecular mechanism of elicitation: Recognition of elicitors by plasma membrane‐bound receptors results in ion fluxes, Ca^2+^ burst, cytoplasmic acidification, ROS burst, NADPH oxidase activation, G‐protein activation and mitogen‐activated protein kinase phosphorylation. It also activates downstream signalling pathway messengers like salicylic acid, jasmonic acid and methyl jasmonate. Messengers activate transcription factors and gene expression, which lead to reprogramming secondary metabolism. [Colour figure can be viewed at wileyonlinelibrary.com]

Signal recognition is mediated by receptors and elicitor‐binding sites present on the plasma membrane in response to elicitors, which activate the next cascade of events like ion fluxes, Ca^2+^ burst, cytoplasmic acidification, ROS burst, NADPH oxidase activation, G‐protein activation and MAPK phosphorylation.^31^ The initial plant response is the exchange of ions, for instance K^+^/Cl^−^ effluxes and Ca^2+^/H^+^ influxes, in response to elicitors. Ca^2+^ influx is considered as the most important event because of its diverse involvement in physiological and cellular processes.^48^, ^49^ Ca^2+^ signals are elaborated through conformational changes in various Ca^2+^‐binding proteins such as calmodulin, calmodulin‐like proteins, calcium‐dependent kinases (CDPKs) and phospholipases as well as through secondary messengers like inositol 1,4,5‐ triphosphate (IP_3_) and diacylglycerol (DAG).^50^, ^51^, ^52^ Ca^2+^/Calmodulin‐mediated pathways are involved in many physiological responses of plants to stimuli. CDPKs have diverse roles in downstream signalling cascades and protein phosphorylation to coordinate cellular processes like regulation of the oxidative burst, hormonal signalling and gene expression.^53^ ROS generation is another important phenomenon in plant defence response, as is the effect of NADPH oxidase and other oxidases in plant cells, and even Ca^2+^ spiking is also responsible for ROS generation.^49^, ^54^, ^55^ Studies have shown the linked role of G‐proteins in stimulating ion channels, phospholipase A, phospholipase C and phospholipase D, ROS generation and cell death in plants.^50^, ^56^, ^57^ Activated G‐protein can stimulate the level of cAMP, IP_3_ and DAG, which triggers the target kinases PKA and PKC. These induced protein kinases cause phosphorylation of MAPKs, which results in gene expression leading to enzymatic reactions, which in turn reprogram the pathway of secondary metabolite production^58^ (Figure 2).

## Manipulation of *Hypericum perforatum* Secondary Metabolism *Via* Elicitation

In general, the type of culture rather than the type of elicitor defines the compounds induced in *H. perforatum* upon elicitation. Hypericins are the most frequently elicitor‐induced compounds in seedlings and shoot cultures, probably due to the presence of hypericin nodules. On the other hand, cell suspensions, calli and root cultures mostly form flavonoids and xanthones.

**Figure 3:**
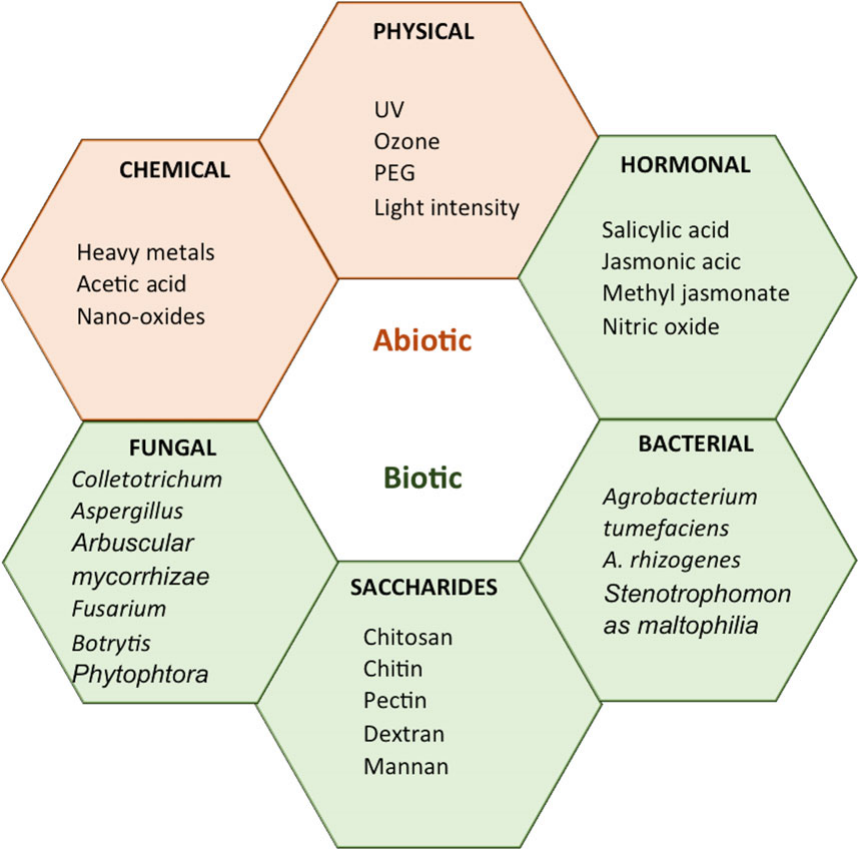
Elicitors of biotic and abiotic origin tested for the induction of secondary metabolites in *Hypericum perforatum*. [Colour figure can be viewed at wileyonlinelibrary.com]

In addition to the type of culture, several other factors affect the success of elicitation, which include the type of elicitor, concentration, incubation conditions and duration of elicitor treatment. Various biotic and abiotic elicitors tested for the manipulation of *H. perforatum* secondary metabolism are categorized in Figure 3. The important compounds induced by these elicitors in various types of *H. perforatum* cultures such as seedling, shoot, root, callus and cell suspension are listed in Table 1.

**Table 1:** Various types of *Hypericum perforatum* cultures and elicitors used in the induction of secondary metabolites

Culture type	Elicitor	Compounds elicited	Reference
Cell suspension	Zinc nano‐oxide	Hypericin and hyperforin	41
	Iron nano‐oxide	Hypericin and hyperforin	41
	Chitin	Phenolics, flavonols, flavanols, anthocyanins, hypericin and pseudohypericin	66
	Pectin	Phenolics, flavonols, flavanols, anthocyanins, hypericin and pseudohypericin	66
	Dextran	Phenolics, flavonols, flavanols, anthocyanins, hypericin and pseudohypericin	66
	*Phytophthora cinnamoni* fungal cell wall extract	None	39
	*Colletotrichum gloeosporioides* mycelium extract	Xanthones and flavonoids	35
	*Aspergillus flavus* mycelium extract	Phenolic compounds, flavanols, flavonols, anthocyanins and hypericins	62
	*Fusarium oxysporum*	Phenolics, flavonoids, anthocyanins, hypericin and pseudohypericin	63
	*Phoma exigua*	Phenolics, flavonoids, anthocyanins, hypericin and pseudohypericin	63
	*Botrytis cinerea*	Phenolics, flavonoids, anthocyanins, hypericin and pseudohypericin	63
	JA	Hypericins	39
	JA	Phenolic compounds, flavanols, flavonols, hypericin and pseudohypericin	38
	MeJA	Flavones	35
	MeJA	Flavonoids	8
	SA	Xanthones	85
	SA	None	35
	SA	None	39
	SA	Hypericins, pseudohypericins	86
	SA	Flavonoids	8
	MeJA +* C. gloeosporioides*	Xanthones	35
	SA +* C. gloeosporioides*	Xanthones	35
	*Agrobacterium tumefaciens*	Xanthones	71
	*A. tumefaciens*	Lignin and flavanoids	72
	*A. tumefaciens*	Phenolics, flavonols, flavanols and xanthones	73
	*A. rhizogenes*	Phenolics, flavonols, flavanols and xanthones	73
Callus	SA	Hypericins, pseudohypericins	86
Root	MeJA	Hypericin	84
	IBA	Hypericin	84
	Acetic acid	Xanthones	91
	Chitosan	Xanthones	68
	Chitosan	Xanthones	68
Shoot	SA	Hypericins and pseudohypericins	86
	Mannan	Pseudohypericin and hypericin	59
	*β*‐1,3‐glucan	Pseudohypericin	59
	Pectin	Pseudohypericin	59
	Yeast extract	None	59
	Chitin	None	64
	Pectin	Hypericin and pseudohypericin	64
	Dextran	Hypericin and pseudohypericin	64
	Saccharose	Hypericin and hyperforin	69
	Saccharose + PEG	Hypericin and hyperforin	69
	Saccharose + MeJA	Hypericin and hyperforin	69
	Saccharose + *A. tumefaciens*	Hypericin and hyperforin	69
	*Stenotrophomonas maltophilia* culture filtrate	Pseudohypericin	70
Seedling	Chromium	Protopseudohypericin, hypericin and pseudohypericin	40
	Nickel	None	12
	*Rhizophagus intraradices*	Hypericin and pseudohypericin	60
	*S. maltophilia*	Hypericin and pseudohypericin	70

SA, salicylic acid; JA, jasmonic acid; MeJA, methyl jasmonate; PEG, polyethylene glycol.

### Biotic elicitation

#### Fungi and extracts from fungal mycelia

In *H. perforatum* shoot cultures, yeast extract did not show any stimulatory effect on either hypericin or pseudohypericin production.^59^ The arbuscular mycorrhizal fungi (AMF) species *Rhizophagus intraradices* enhanced *H. perforatum* seedling growth and hypericin and pseudohypericin production, when supplied to the soil either alone or as mixture with AMF, namely *Funneliformis constrictum, F. geosporum* and *F. mosseae*.^60^

*Hypericum perforatum* cell suspension cultures treated with *Colletotrichum gloeosporioides* cell wall extract showed a significant increase in xanthone accumulation.^35^ Interestingly, this xanthone accumulation was increased 12‐fold when the cultures were primed with MeJA or SA, before adding the fungal extract. *Aspergillus niger*,* Fussarium oxysporum* and yeast extracts increased the accumulation of phenolic compounds and flavonoids in addition to the production of new constituents such as *p*‐hydroxybenzoic acid in the suspended cells of *H. triquetrifolium*.^61^ On the other hand, mycelium extract from *Aspergillus flavus* only stimulated the level of anthocyanins in *H. perforatum* cell suspensions.^62^

Interestingly, cell wall extracts from *F. oxysporum*,* Phomaexigua* and *Botrytis cinerea* showed rapid stimulation of naphthodianthrones (hypericin and pseudohypericin) in addition to flavonoids and anthocyanins in cell suspension cultures of *H. perforatum*.^63^ *A. niger* cell walls induced hypericin biosynthesis in *H. perforatum* cell suspension cultures.^36^

#### Polysaccharides

Oligosaccharides and polysaccharides, respectively, from fungal and plant cell walls are the most studied signalling molecules in elicitation pathways.^31^ In *H. perforatum* shoot cultures, mannan, *β*‐1,3‐glucan, pectin and yeast extract were studied for the production of naphthodianthrones.^59^ Although mannan stimulated the production of pseudohypericin and hypericin substantially, *β*‐1,3‐glucan and pectin showed a weak effect on pseudohypericin production but had no effect on hypericin production. While pectin and dextran enhanced the content of pseudohypericin and hypericin, chitin did not show any stimulatory effect in *H. perforatum* shoot cultures.^64^ Mannan and pectin were tested for the biosynthesis of hypericins in *H. adenotrichum* seedlings grown *in vitro*.^65^ Although both elicitors stimulated the biosynthesis of pseudohypericin and hypericin, the stimulatory potential of mannan was lower than that of pectin.

Treatment with chitin, pectin and dextran improved production of phenylpropanoids (phenolics, flavanols, anthocyanins) in cell suspension cultures.^66^ Brasili et al.^67^, ^68^ studied the effect of chitosan treatment in *H. perforatum* adventitious root cultures. Chitosan increased the synthesis of epicatechin, xanthones and isoprenoids^68^ and a new xanthone, brasilixanthone B, was identified in treated root cultures.^67^

#### Bacteria

Elicitation of *H. perforatum* shoot cultures with a combination of saccharose and inactivated *Agrobacterium tumefaciens* promoted the hypericin and hyperforin production.^69^ Treatment of *H. perforatum* seedlings with *Stenotrophomonas maltophilia* increased the hypericin and pseudohypericin contents.^70^ These authors also reported that treating *H. perforatum* shoot cultures with a *S. maltophilia* culture filtrate induced only pseudohypericin production.^70^

Analysis of the methanolic extract of cell suspension cultures treated with *A. tumefaciens* revealed a 12‐fold increase in the total xanthone concentration and the emergence of many new xanthones.^71^ In addition, the contents of lignin and flavonoids (quercetin, quercetrin etc.) were also significantly increased in the cell wall fraction of phenolics after elicitation with *A. tumefaciens*.^72^ Similarly, improvement of phenolic, flavonol, flavanol and xanthone concentrations in response to *A. tumefaciens* and *A. rhizogenes* treatment was observed in cell suspension cultures.^73^ However, *A. rhizogenes* was less effective in the induction of secondary metabolism compared to *A. tumefaciens*.^73^ Induction of *H. perforatum* plant secondary metabolism by *Agrobacterium* might be attributed to components such as cold‐shock protein, flagellin, peptidoglycan and elongation factor‐Tu.^74^, ^75^, ^76^, ^77^, ^78^, ^79^, ^80^

#### Signalling compounds and plant growth regulators

Plant defence signalling compounds such as SA, MeJA, JA have been shown to modulate *H. perforatum* secondary metabolism. When treated with an analogue of MeJA, 2,3‐dihydroxypropyl jasmonate, hypericins and hyperforin production was increased in *H. perforatum* and *H. sampsonii* shoot cultures.^81^ Different combinations of plant growth regulators and signalling compounds (JA and SA) enhanced the accumulation of hypericins, pseudohypericin and hyperforin in shoot cultures of *H. hirsutum* and *H. maculatum*.^82^ The supplementation of plant growth regulators in culture medium variously affected naphthodianthrone accumulation in shoot cultures.^83^ This study reported that the presence of IBA actually decreased the concentration of hypericin and pseudohypericin although the naphthodianthrone production in plantlets was not influenced by IAA supplementation.

The MeJA concentration showed a negative correlation with biomass production and a positive correlation with hypericin production in adventitious root cultures grown in balloon‐type airlift bioreactors, in which an optimum hypericin content of 1.61 mg/g DW was achieved at 350 μm MeJA.^84^

The effect of signalling compounds on the induction of secondary metabolism in cell suspension cultures of *H. perforatum* is divergently reported between groups. Conceicao *et al*.^35^ reported that MeJA‐treated *H. perforatum* cell suspension cultures accumulated new flavonoids (flavones), whereas SA was unable to induce any change of secondary metabolism. Elicitation of cell cultures with 100 μmol/l MeJA on day 15 resulted in 2.7 times higher flavonoid production (280 mg/l) compared to control cultures.^8^ Another study reported that JA treatment significantly increased phenolic compounds, flavanols and flavonols, concomitantly reducing the anthocyanin content.^38^ A twofold increase in the xanthone, cadensin G and paxanthone contents was found in cell suspension cultures of *H. perforatum* following elicitation with SA.^85^

Walker *et al*.^39^ found that JA dramatically enhanced hypericin production in cell cultures incubated in the dark compared to cultures grown under photoperiodic conditions, although SA treatment could not induce hypericin production in cell suspensions. Contrarily, SA remarkably influenced the production of hypericin and pseudohypericin in *H. perforatum* cell suspension cultures as per the report of Gadzovska‐Simic *et al*.^86^

### Abiotic elicitation

#### Physical factors

A few physical factors such as exposure to high light intensity, ozone, UV and osmotic stress have been tested for the induction of secondary metabolism in *H. perforatum*. The hyperforin, pseudohypericin and hypericin concentrations were altered in *H. perforatum* plants after UV‐B exposure during the vegetative growth.^87^ However, the level and length of UV‐B radiation negatively affected the hypericin concentration in leaves, although the flavonoid and tannin contents increased with enhanced levels of UV‐B exposure.^88^ Treatment with polyethylene glycol (PEG) increased the hypericin and pseudohypericin concentrations in cultured *H. adenotrichum* seedlings. However, this increase was dependent on the concentration of PEG and the treatment period, 10 g/l PEG for a 15‐day treatment being optimal.^89^ On the other hand, synthesis of hypericin and hyperforin in *H. perforatum* shoot cultures was not increased in the presence of 10 and 15 g/l PEG, although a lower PEG concentration stimulated the production of those compounds.^69^ Sucrose as an osmotic agent was shown to stimulate hypericins in cultured seedlings of *H. adenotrichum* with increasing concentrations until 45 g/l, followed by decline.^89^ Correspondingly, the presence of saccharose (10–30 g/l) influenced the production of hypericin and hyperforin in *H. perforatum* shoot cultures and a combination of PEG (1.25–5 g/l) and saccharose (10–30 g/l) synergistically increased the production of the above compounds.^69^

*Hypericum perforatum* cell suspension cultures accumulated anthocyanins and flavonoids with concomitant reduction of xanthones upon increasing the irradiance of cultures from 20 μmol/s per m^2^ to 60 μmol/s per m^2^.^90^ Exposure to ozone stimulated hypericin synthesis in *H. perforatum* cell suspension cultures, with the cell cultures at the exponential phase being more responsive than the ones at the lag and stationary phases.^91^

#### Chemical

Chromium treatment induced the production of protopseudohypericin, hypericin and pseudohypericin in *H. perforatum* seedlings, whereas nickel treatment did not show any stimulatory effect.^40^ In *H. perforatum* root cultures, acetic acid enhanced xanthone production.^91^

Treatment of *H. perforarum* cell suspension cultures with 100 ppb zinc and iron nano‐oxides promoted hypericin and hyperforin production, while higher concentrations of these nanomaterials negatively affected the production of these compounds.^41^

### Pharmacological properties of *Hypericum perforatum* after elicitation

Although a number of studies report the elicitor‐induced modulation of the phytochemical profile of *H. perforatum in vitro* cultures, only a few of them actually tested the bioactivity of the treated cultures. The methanolic extract of *H. perforatum* cell suspension cultures challenged with *A. tumefaciens* was studied for various bioactivities. This extract showed a higher antioxidant potential, a greater capacity to prevent synaptosomal lipid peroxidation^71^ and improved antimicrobial activity against several human pathogenic bacteria.^92^ It also offered human HepG2 cells more protection against t‐BOOH‐induced oxidative damage, compared to control extract.^93^ Tocci *et al*.^94^ have shown that extracts of root tissue elicited with chitosan, *O*‐carboxymethyl chitosan and its derivatives showed antifungal activity against human fungal pathogens such as *Candida* spp., *Cryptococcus neoformans* and dermatophytes.

## Future Perspectives

### Potential application of nanotechnology

In addition to several well‐known biotic and abiotic elicitors discussed above, nanoparticles are currently emerging as new class of elicitors. A few studies on the induction of secondary metabolites in response to nanoparticle treatment provide insights in the exploitation of these submicron size particles as elicitors of secondary metabolites. For instance, the artemisinin content was increased in a hairy root culture of *Artemisia annua* upon treatment with core–shell silver nanoparticles.^95^ In *Calendula officinalis*, the anthocyanin and flavonoid contents were decreased, whereas carotenoid and saponin contents were increased in response to silver nanoparticles, as revealed by classical colorimetric assays.^96^ In addition, multiwalled carbon nanotubes stimulated the biosynthesis of secondary metabolites and the antioxidant capacity in the medicinal plant *Satureja khuzestanica* grown *in vitro*.^97^ In the case of *H. perforatum*, one study has reported that zinc and iron nano‐oxides stimulate hypericin and hyperforin production in cell suspension cultures.^41^

In addition to the use of metallic nanoparticles as elicitors, signalling compounds such as JA, SA and MeJA can be encapsulated in biodegradable polymers as nanoparticles and can be exploited for sustained release of the signalling molecules into the culture medium, which might be useful in sustained production of secondary metabolites without affecting the growth of cultures. The possibility of using metallic nanoparticles as trappers of secondary metabolites from plants has been demonstrated in *A. thaliana*.^98^ The authors reported that anatase TiO_2_ nanoparticles (smaller than 20 nm) enter plant cells, conjugate enediol‐ and catechol group‐rich flavonoids *in situ* and exit plant cells as flavonoid‐nanoparticle conjugates. The compound adsorption capacities of nanoparticles may be further improved by functionalization. For instance, the adsorption capacity of SiO_2_ nanoparticles towards quercetin could be improved by TiO_2_ functionalization compared to non‐functionalized and decyl group‐functionalized SiO_2_ nanoparticles due to possible binding of quercetin to the metal oxide.^99^ This adsorption capacity increased linearly with surface coverage of TiO_2_ emphasizing the correlation between the functional surface and quercetin adsorption. This phenomenon may provide us an attractive opportunity to establish nanotrapping strategies for a wide variety of secondary metabolites in the near future.

### Elicitation of *Hypericum perforatum* secondary metabolism: a road to drug discovery

**Figure 4:**
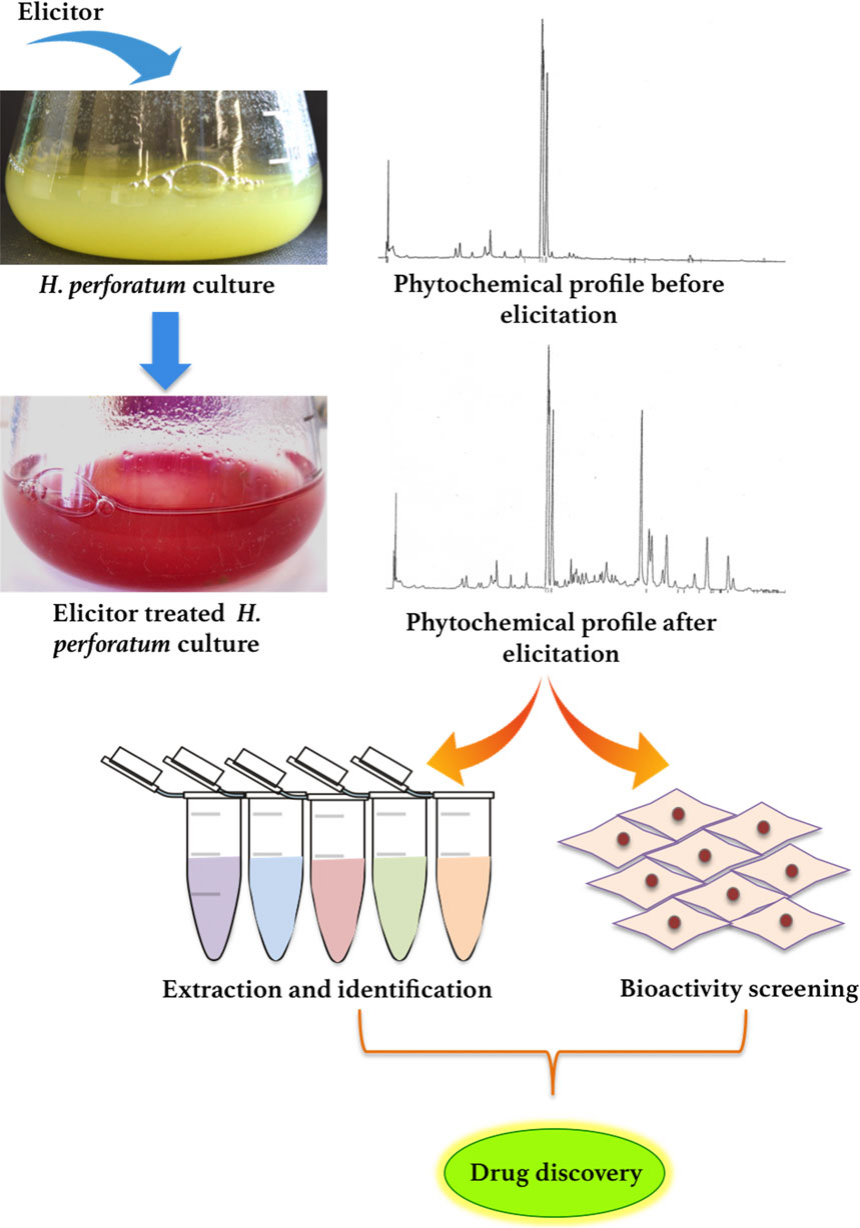
Scheme of possible exploitation of elicitation‐mediated changes in the *Hypericum perforatum* secondary metabolite profile for drug discovery *via* bioactivity‐guided fractionation. [Colour figure can be viewed at wileyonlinelibrary.com]

Although manipulation of *H. perforatum* secondary metabolism *via* elicitation has been reported in numerous studies, very few authors have actually tested the pharmacological properties of induced compounds and extracts from treated cells.^92^, ^93^, ^94^ To promote the discovery of novel drugs, extracts from elicitor‐treated cells containing new compounds should be analysed and screened for a variety of bioactivities. Bioactivity‐guided fractionation may be used. The possible road to drug discovery after elicitation is summarized in the following Figure 4.

In spite of the exciting possibility of inducing diverse classes of bioactive compounds in *H. perforatum* cell and tissue cultures *via* elicitation, industrial exploitation of elicitation‐based changes in secondary metabolism and pharmacological properties is still in its infancy. The production of significant quantities of aseptic biomass for elicitation is the major concern. In this context, small‐scale bioreactors of *in vitro* cultures for the production of active compounds have been reported.^18^ Recently, adventitious root cultures in large‐scale bioreactors for the production of *H. perforatum* phytochemicals have been developed.^84^, ^100^ The choice of the correct culture vessels and the determination of exogenous signals needed for *in vitro* production of biomass and optimization of elicitation measures are essential factors for the further progress in this area.

## Conclusion

Based on our analysis of the literature, it is evident that seedlings and *in vitro* cultures of *H. perforatum* are efficient systems to produce a wide variety of bioactive secondary metabolites *via* elicitation. The paradoxical results obtained between groups in terms of phytochemical profile, namely hypericin production after elicitation, argue for the complexity of culture maintenance, elicitation and analytical methods. For example, under normal conditions, hypericin is produced only in the presence of dark hypericin nodules. Several studies have confirmed a clear positive correlation between the presence of dark cell clusters and hypericin accumulation irrespective of the tissue, genotype, species etc.^101^, ^102^, ^103^, ^104^ Roots of *H. perforatum* plants lack these clusters, where no traces of hypericin were found.^105^ In spite of the absence of these nodules in roots and cell suspension cultures of *H. perforatum*, few groups have actually reported the formation of hypericins in these cultures after elicitation.^38^, ^39^, ^41^, ^63^, ^66^, ^84^, ^86^ Further investigation on the ability of *H. perforatum* cell and tissue cultures without these dark cell clusters to produce hypericin might offer novel clues on the hypericin biosynthetic pathway.

As the secondary metabolite elicitation potential varies between the types of cultures, elicitors, treatment conditions and other parameters, further research is needed to optimize the best and reproducible protocols. In this context, understanding the metabolic pathways leading to the production of specific secondary metabolites and their regulation is imperative. However, with the exception of the flavonoid pathway, complex biosynthetic pathways specifically evolved in *Hypericum* spp. are far from being understood. The lack of relevant information on the enzymes and genes involved as well as the transcription factors and master switches controlling these pathways is the major obstacle for developing efficient strategies for elicitation of *H. perforatum* secondary metabolism. Comparative transcriptomic, metabolomic and proteomic studies carried out on both control and elicitor‐treated cultures are expected to offer important clues.

## Declarations
